# Decreased microbiome diversity in the HIV small airway epithelium

**DOI:** 10.1186/s12931-018-0835-7

**Published:** 2018-07-27

**Authors:** Stella Xu, Amy Tsai, Marc A. Sze, Emily A. Vucic, Tawimas Shaipanich, Marianne Harris, Silvia Guillemi, Julia Yang, Sunita Sinha, Corey Nislow, Julio Montaner, Wan Lam, Stephen Lam, Don D. Sin, S. F. Paul Man, Janice M. Leung

**Affiliations:** 1 Centre for Heart Lung Innovation, University of British Columbia, Vancouver, BC Canada; 2 Department of Medicine, Faculty of Medicine, University of British Columbia, Vancouver, BC Canada; 3 Department of Microbiology and Immunology, University of Michigan, Ann Arbor, MI USA; 4 British Columbia Cancer Agency, Vancouver, BC Canada; 5 Division of Respiratory Medicine, St. Paul’s Hospital, Vancouver, BC Canada; 6 British Columbia Centre for Excellence in HIV/AIDS, St. Paul’s Hospital, Vancouver, BC Canada; 7 Faculty of Pharmaceutical Sciences, University of British Columbia, Vancouver, BC Canada; 8 Centre for Heart Lung Innovation, St. Paul’s Hospital, Room 166-1081 Burrard Street, Vancouver, V6Z 1Y6 Canada

**Keywords:** HIV, Microbiome, Epithelium, COPD

## Abstract

**Background:**

Persons living with human immunodeficiency virus (PLWH) face an increased burden of chronic obstructive pulmonary disease (COPD). Repeated pulmonary infections, antibiotic exposures, and immunosuppression may contribute to an altered small airway epithelium (SAE) microbiome.

**Methods:**

SAE cells were collected from 28 PLWH and 48 HIV- controls through bronchoscopic cytologic brushings. DNA extracted from SAE cells was subjected to 16S rRNA amplification and sequencing. Comparisons of alpha and beta diversity between HIV+ and HIV- groups were performed and key operational taxonomic units (OTUs) distinguishing the two groups were identified using the Boruta feature selection after Random Forest Analysis.

**Results:**

PLWH demonstrated significantly reduced Shannon diversity compared with HIV- volunteers (1.82 ± 0.10 vs. 2.20 ± 0.073, *p* = 0.0024). This was primarily driven by a reduction in bacterial richness (23.29 ± 2.75 for PLWH and 46.04 ± 3.716 for HIV-, *p* < 0.0001). Phyla distribution was significantly altered among PLWH, with an increase in relative abundance of Proteobacteria (*p* = 0.0003) and a decrease in Bacteroidetes (*p* = 0.0068) and Firmicutes (*p* = 0.0002). Six discriminative OTUs were found to distinguish PLWH from HIV- volunteers, aligning to Veillonellaceae, *Fusobacterium*, Verrucomicrobiaceae*, Prevotella, Veillonella,* and *Campylobacter.*

**Conclusions:**

Compared to HIV- controls, PLWH’s SAE microbiome is marked by reduced bacterial diversity and richness with significant differences in community composition.

**Electronic supplementary material:**

The online version of this article (10.1186/s12931-018-0835-7) contains supplementary material, which is available to authorized users.

## Background

For the 35 million people living with human immunodeficiency virus (PLWH), antiretroviral therapy (ART) has been a lifesaving measure. As a result, AIDS-related morbidity and mortality have decreased markedly; however, aging with HIV has brought other challenges [1]. For instance, PLWH are more likely to develop chronic obstructive pulmonary disease (COPD) [2] and are also more likely to suffer from severe respiratory symptom burdens even after adjustment for smoking habits [3]. Although the pathogenesis of accelerated COPD in PLWH is poorly understood, the unique risk for pulmonary infections in this setting suggests that shifts in the lung microbiome might account for this phenomenon.

Investigations into the HIV lung microbiome have yielded interesting insights but no clear consensus. Lozupone et al. found that the abundance of *Tropheryma whipplei* was significantly increased in bronchoalveolar lavage (BAL) samples of PLWH compared with HIV- control subjects [4], while a second study by Beck et al. showed no differences between the two groups [5]. A third study also evaluating BAL demonstrated that PLWH who had advanced disease (CD4 cell counts < 500 cells/mm^3^) had significantly reduced microbiome diversity when compared to HIV- controls, with diversity starting to return to normal levels once ART was initiated [6]. While these studies have offered the first insights into the HIV lung microbiome, the reliance on BAL fluid may fail to identify important changes at the specific initial site of injury in the pathogenesis of COPD, namely the small airway [7]. The small airway epithelium (SAE) is the first line of defense against toxins such as cigarette smoke and microbial pathogens. In COPD, remodeling of this layer with squamous metaplasia, goblet cell hyperplasia, and breakdown of the epithelial barrier junction are critical to injury development [8]. Moreover, evidence that endotoxins produced by *Staphylococcus aureus* and *Haemophilus influenza* can damage epithelial barrier function suggests that an important relationship between the microbiome, epithelial injury, and COPD may exist [9]. Previous work by our group identified that within PLWH, the absence of Pasteurellaceae and *Brachybacterium* and the presence of *Yersinia* species in the SAE could help identify those with COPD [10]. Our study explores whether significant differences exist between the SAE microbiomes of PLWH and uninfected controls.

## Methods

### Study cohort

PLWH were drawn from the patient population at St. Paul’s Hospital in Vancouver, Canada, a tertiary care setting with an active bronchoscopy program and HIV outpatient clinic. Eligible PLWH were patients who were undergoing bronchoscopies for clinical purposes (i.e. for lung masses or nodules or to rule out infection) and were consented for additional research specimen collection during the procedure. All subjects were ≥ 19 years old and provided written informed consent under the University of British Columbia (UBC) Providence Health Care ethics protocol H14–03267. HIV- controls were recruited from patients undergoing lung cancer screening bronchoscopies at the British Columbia Cancer Agency in Vancouver, Canada. With the exception of seven HIV-infected patients who were lost to follow-up, all subjects underwent pre-bronchodilator spirometry according to guidelines provided by the American Thoracic Society and European Respiratory Society [11]. COPD was defined according to criteria outlined by the Global Initiative for Chronic Obstructive Lung Disease (GOLD) [12].

### Sample collection

SAE cells were obtained via bronchoscopic cytologic brushings. Samples were obtained prior to the collection of clinical specimens and away from sites of disease as detected by chest computed tomography (CT) imaging performed within a month of the bronchoscopy. The bronchoscope was inserted in the oral cavity into the trachea and bronchi with minimal use of the suction channel to avoid contamination. Cytologic brushes were then directed in the subsegment of interest until resistance was encountered (in the 5th and 6th generation airways). Brushings were taken at that site and collected in Cytolyt (Cytyc, Marlborough, MA) for DNA preservation.

### DNA extraction and PCR amplification

DNA was extracted using the Qiagen DNeasy Blood and Tissue Kit (Qiagen, Toronto, Ontario). Samples were eluted with 50 ul of distilled water and the DNA concentration was measured by NanoDrop (ThermoFisher Scientific, Waltham, MA). All samples were normalized to 12 ng/ul for downstream experiments. To quantify total bacteria load in each sample, primers specifying the 293 bp amplicon of the 16S rRNA gene were designed using the protocol outlined by Sze *et a.l* [13]. A pooled library consisting of all the samples with individually labeled indices was generated using the protocol adopted from a dual-index sequencing strategy published by Kozich et al. [14]. One exception to this protocol was that touchdown PCR was used to amplify the 16S rRNA gene fragments spanning the V4 region. PCR products were purified with Agencourt AMPure XP system (Beckman Coulter, Catalog #A63880). Sequencing was performed on the Illumina MiSeq™ platform (Illumina, Redwood City, CA, USA) with 2 × 250 paired end-read chemistry at the UBC Sequencing and Bioinformatics Consortium. Further details regarding the PCR amplification are provided in the supplement.

### Microbiome profiling

Sequencing reads were merged, filtered for quality, and processed using the software mothur v.1.35.1 [15] according to the Standard Operating Procedure for MiSeq data (http://www.mothur.org). The accepted sequences were clustered into operational taxonomic units (OTUs) using the 97% identity threshold, and classified using the Ribosomal Database Project (RDP) Classifier [16] and the RDP taxonomy training set (http://rdp.cme.msu.edu). To account for potential sources of contamination, OTUs observed in the negative extraction controls (sterile water processed along with samples) were considered contaminants and removed from downstream analysis.

### Statistical analysis

Alpha diversity measures (Richness, Shannon diversity index, and Evenness) [17, 18] were obtained using the Vegan package (V2.3.0) [19] in R (V3.2.0), available at https://www.r-project.org, and compared according to COPD or HIV status using the Mann-Whitney U test [18]. Beta diversity analyses based on Bray-Curtis distance matrix were performed, and differences in microbial communities between groups were tested by Permutational Multivariate Analysis of Variance (PERMANOVA) [20]. Nonmetric Multidimensional Scaling [NMDS] was employed as ordination method for visualization of Bray-Curtis dissimilarity values in both groups [21]. Comparisons of relative taxa abundance (at the phylum level) between groups were performed using the Mann-Whitney U test [18]. To identify the most discriminating OTUs between groups, we employed a random forest algorithm with Boruta feature selection [22, 23], and the Vegan package was used to construct abundance heatmaps.

## Results

### Study cohort

Demographic details of the study cohort are provided in Table 1. 28 PLWH (7 with COPD and 14 without COPD, 7 with unknown status) and 48 HIV- controls (24 with COPD and 24 without COPD) were enrolled. Males accounted for the majority of the HIV+ cohort (86%) compared to only half of the HIV- cohort. There were no statistically significant differences in lung function and in smoking status between the two cohorts. Nine (32%) of the PLWH had a detectable plasma HIV viral load and the mean CD4 count for this group was 419 cells/mm^3^. Only four (14%) of the PLWH cohort were not currently on ART.

**Table 1:** Study Cohort Characteristics

	PLWH (*n* = 28)	HIV- (*n* = 48)	*p*-value
Sex			
Male (%)	24 (86%)	25 (52%)	0.003
Female (%)	4 (14%)	23 (48%)	
Age ± SD^a^ (years)	57.54 ± 11.85	63.00 ± 7.65	0.038
FEV1/FVC (%) ± SD^a^	70.01 ± 12.62	68.39 ± 15.24	0.498
Smoking Status			
Current (%)	12 (43%)	21 (44%)	0.988
Past (%)	14 (50%)	24 (50%)	
Never (%)	2 (7%)	3 (6%)	
HIV Plasma Viral Load > 50 copies/mL (%)	9 (32%)	N/A	N/A
CD4 cell count ± SD^a^ (cells/mm^3^)	419 ± 295	N/A	N/A

^a^*SD* Standard deviation

### Total bacterial load

Total 16S rRNA (reflective of total bacterial loads) was significantly reduced in PLWH compared to the HIV- cohort (0.27 ± 1.10 copies/ng DNA vs. 3.25 ± 8.44 copies/ng DNA, *p* = 0.0076). However, there were no differences in total 16S rRNA between HIV+ patients with and without COPD (*p* = 0.653) or between HIV- controls with and without COPD (*p* = 0.719).

### Bacterial diversity

**Fig. 1:**
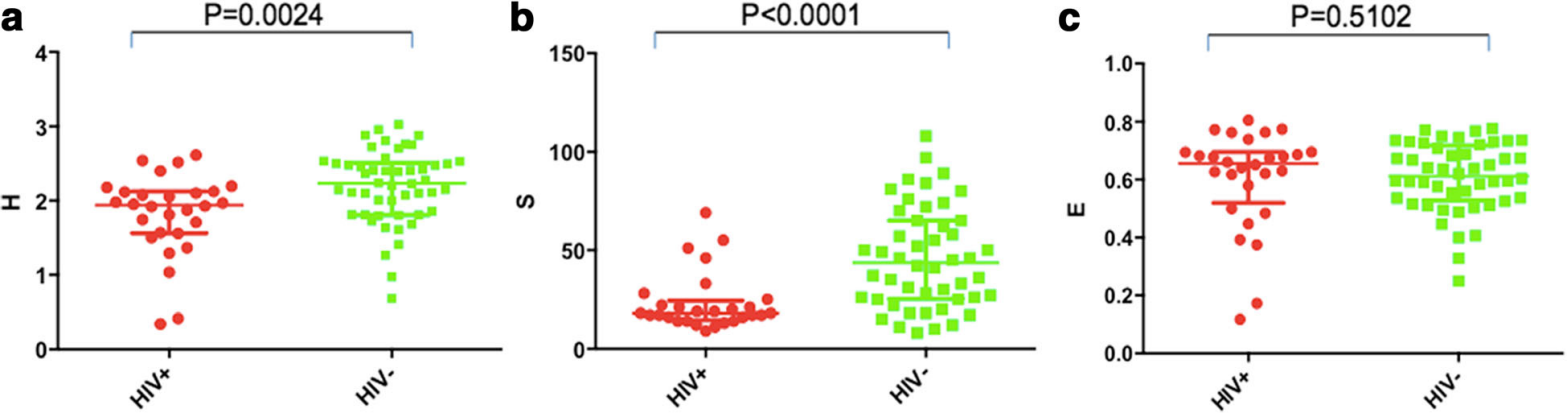
Shannon diversity **a**, richness **b**, and evenness **c** are shown for PLWH (red) and HIV- subjects (green). Shannon diversity and richness were both significantly lower in PLWH compared with HIV- subjects. There was no significant difference in species evenness between the two groups

Alpha diversity was assessed using the Shannon diversity index, richness, and evenness (Fig. 1). There was a significant decrease in Shannon diversity in the HIV+ compared to the HIV- groups (1.82 ± 0.10 vs. 2.20 ± 0.073, *p* = 0.0024), driven mainly by a significant reduction in bacterial richness (23.29 ± 2.75 for HIV+ and 46.04 ± 3.716 for HIV-, *p* < 0.0001). The difference in bacterial richness (i.e. overall species count) was confirmed in a rarefaction curve in which more species were found in the HIV- group for each random sample compared with the HIV+ group (Additional file 1: Figure S1). There was no significant difference in evenness between the two groups (0.60 ± 0.032 for HIV+, 0.61 ± 0.017 for HIV-, *p* = 0.5102). Across the four groups (HIV + COPD+, HIV + COPD-, HIV-COPD+, and HIV-COPD-), there were significant differences in Shannon diversity (*p* = 0.0195) and richness (*p* = 0.0006) by Kruskal-Wallis tests. However, this was entirely driven by differences between the HIV+ and HIV- groups as there were no significant differences observed between the COPD+ and COPD- groups. There was also no significant difference in evenness between the four groups (*p* = 0.38). There was a significant direct correlation between FEV1/FVC and Shannon diversity (*p* = 0.0095, *R* = 0.39) in the HIV- group but not within the HIV+ group (*p* = 0.1427, *R* = 0.34). To ensure that the differences between HIV+ and HIV- groups was not confounded by the clinical indication for bronchoscopy within the HIV+ group, we compared Shannon diversity and beta diversity by indication for bronchoscopy, use of inhaled corticosteroids, cancer diagnosis, and treatment or prophylactic antibiotics. No significant differences were found in any of these categories (Additional file 1: Figures S2–S6). There was no correlation between Shannon diversity and CD4 count, nor was there a difference between those with detectable and undetectable viral loads.

### Phyla distribution

**Fig. 2:**
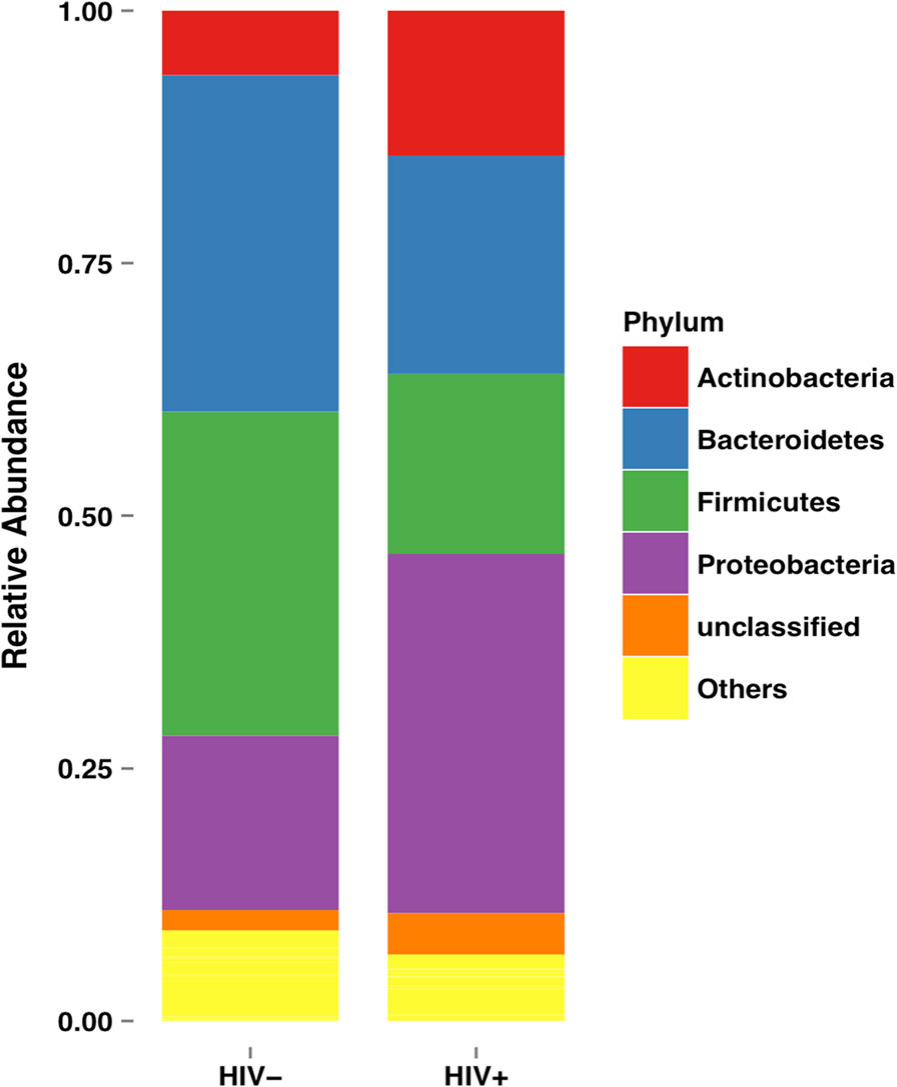
The relative distribution of phyla between PLWH and HIV- patients is shown. There was a significant increase in Proteobacteria (*p* = 0.0003) and decrease in Bacteroidetes (*p* = 0.0068) and Firmicutes (*p* = 0.0002) in PLWH compared to HIV- patients

**Fig. 3:**
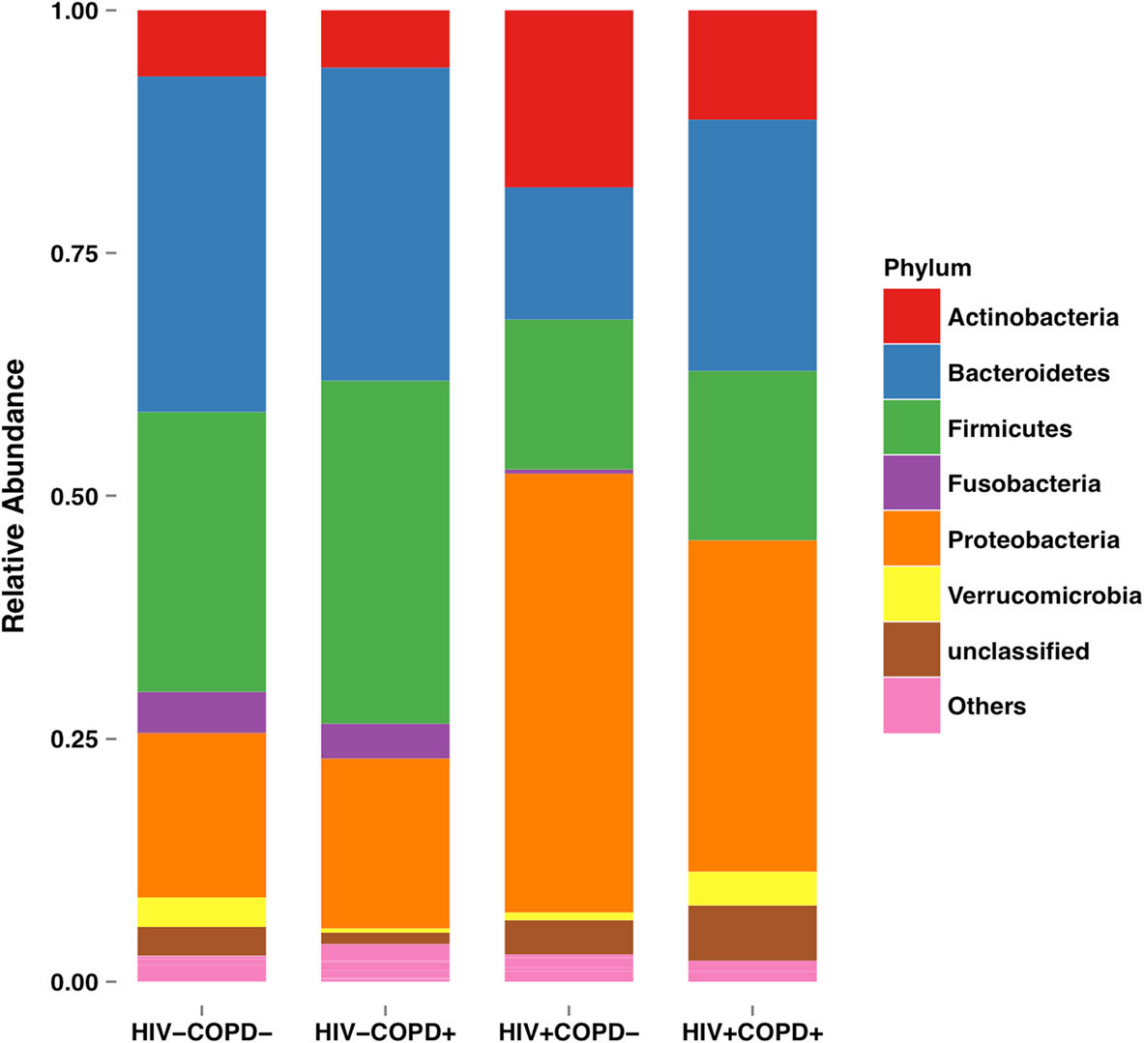
The distribution of phyla across all four groups is shown. By Kruskal-Wallis tests, there were significant differences between the groups in Bacteroidetes (*p* = 0.0031), Proteobacteria (*p* = 0.0013), and Firmicutes (*p* = 0.0017). Post-hoc tests by Dunn’s Multiple Comparison method demonstrated that the differences observed in Bacteroidetes and Proteobacteria were driven entirely by the difference between the HIV + COPD- and HIV-COPD- groups. However, the difference observed in Firmicutes was driven by the difference between the HIV + COPD+ and HIV-COPD+ groups. There were no significant differences in phyla distribution between the COPD+ and COPD- groups

A significant increase was observed in the relative abundance of Proteobacteria in PLWH (0.38 ± 0.25 in HIV+ vs. 0.19 ± 0.19 in HIV-, *p* = 0.0003) (Fig. 2). Decreased abundance of Bacteroidetes (0.23 ± 0.19 in HIV+ vs. 0.35 ± 0.16 in HIV-, *p* = 0.0068) and Firmicutes (0.18 ± 0.17 in HIV+ vs. 0.33 ± 0.16 in HIV-, *p* = 0.0002) were found in the HIV+ group compared to the HIV- group. Figure 3 shows the distribution of phyla across all four patient groups. According to a Kruskal-Wallis test, there was a significant difference in Bacteroidetes (*p* = 0.0031), Proteobacteria (*p* = 0.0013) and Firmicutes (*p* = 0.0017) across the four groups. Post-hoc tests were performed using Dunn’s Multiple Comparison method; the differences observed in Bacteroidetes and Proteobacteria were driven by the difference between the HIV+ and HIV- groups in the COPD- population. The difference observed in Firmicutes was driven by the difference between the HIV+ and HIV- groups in the COPD+ population. There was no significant difference between COPD+ patients and COPD- patients, irrespective of HIV status.

### Microbial compositional analysis

**Fig. 4:**
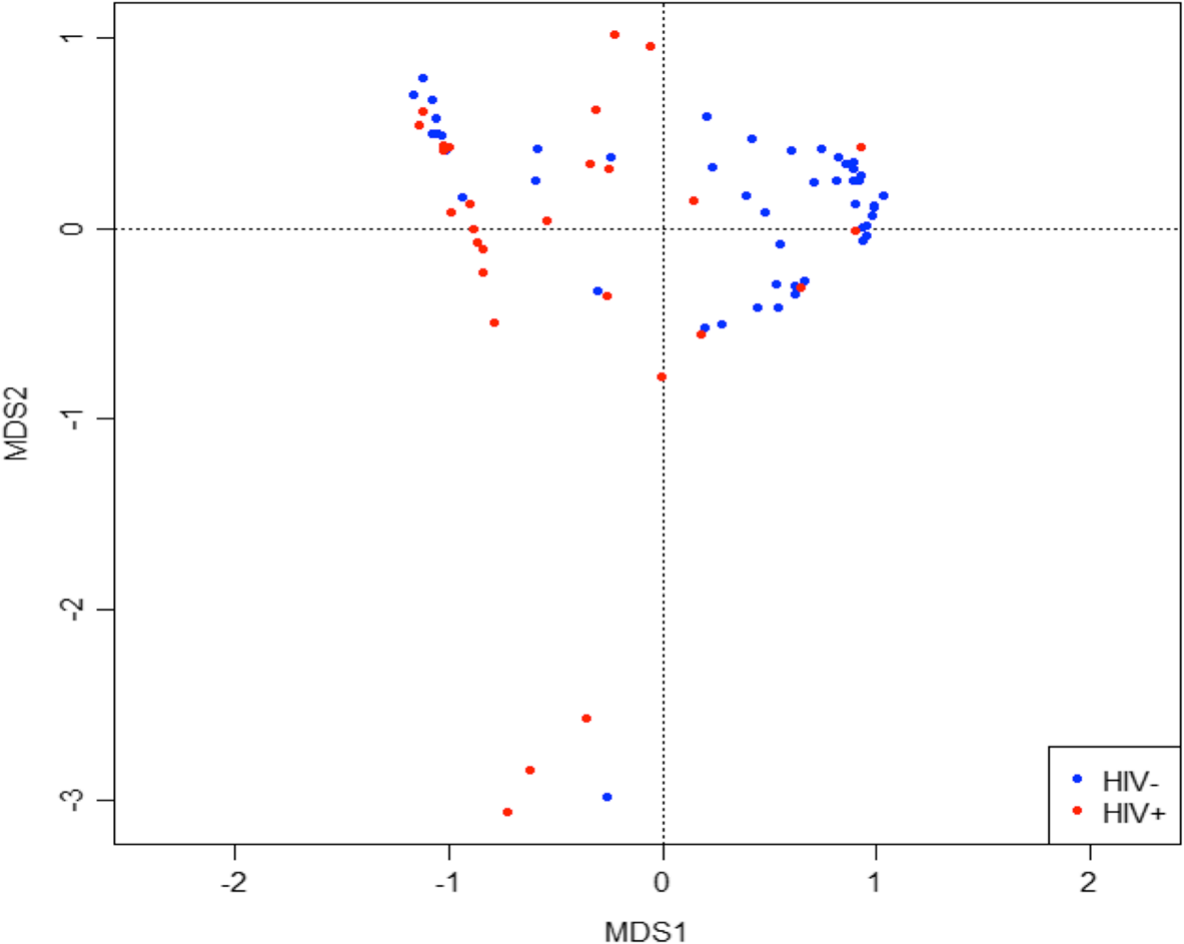
Non-metric multidimensional scaling analysis demonstrates a significant difference in bacterial community composition between PLWH (red) and HIV- subjects (blue) (PERMANOVA = 0.001)

**Fig. 5:**
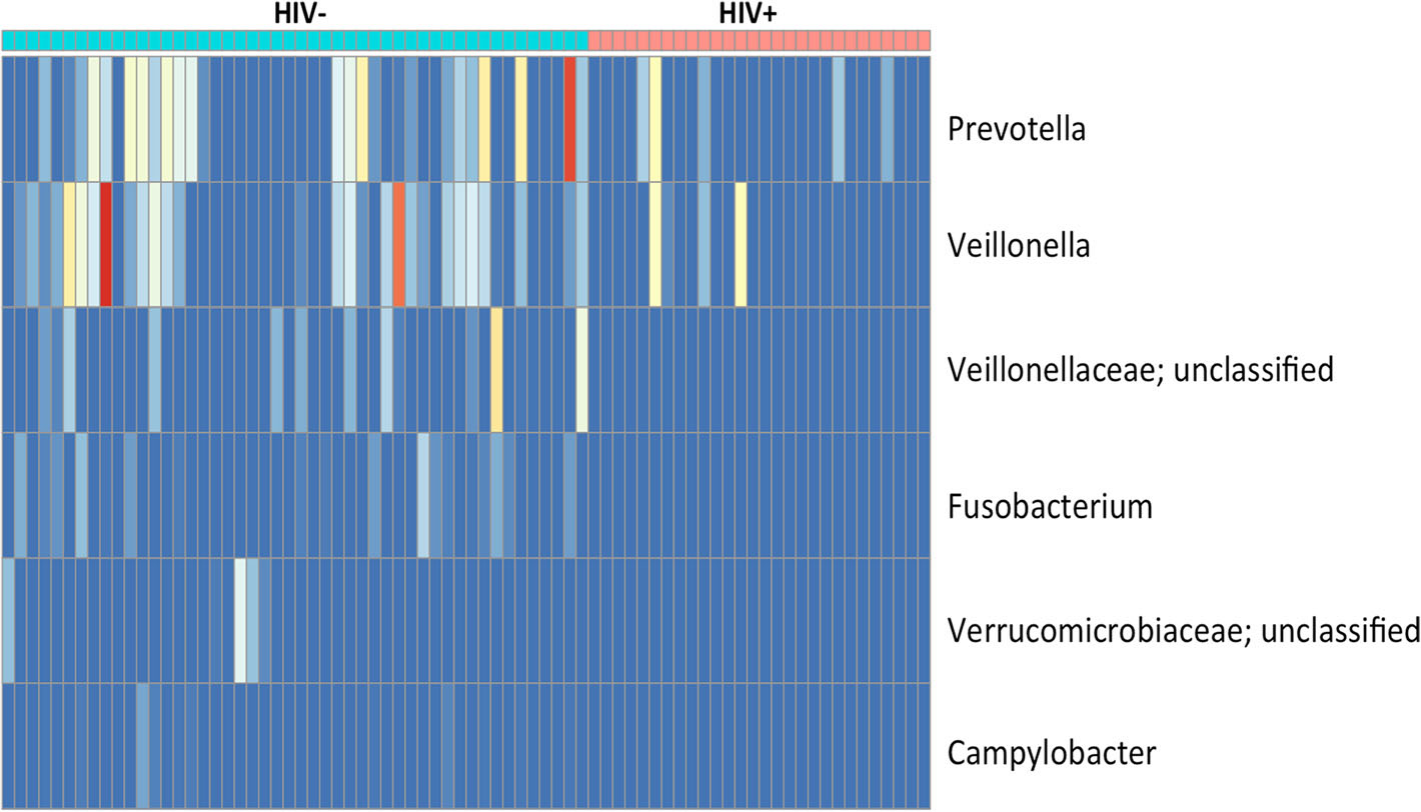
In this heat map of OTU abundance, blue indicates a complete absence of the OTU, yellow a lower relative abundance, and red a higher relative abundance. Six OTUs were found to help distinguish between PLWH and HIV- subjects

Because there was no difference in alpha diversity between the COPD+ and COPD- groups, beta diversity analysis was restricted to comparing the study cohort by HIV status. Using non-metric multidimensional scaling analysis, a significant difference in community composition was detected between the HIV+ and HIV- groups (PERMANOVA = 0.001) (Fig. 4). Using Boruta feature selection with Random Forest analysis, six OTUs were found to be able to discriminate PLWH from the HIV- group. Figure 5 is a heatmap with yellow representing a lower relative abundance, red representing a higher relative abundance and blue representing samples that did not contain such particular OTUs. OTUs that aligned to *Veillonellaceae*, *Fusobacterium*, *Verrucomicrobiaceae* and *Campylobacter* were not found in PLWH. Moreover, *Prevotella* and *Veillonella* were mainly present in HIV- group. These findings suggest that these six discriminative OTUs were able to separate PLWH from the HIV- group.

## Discussion

In this first comparison of the HIV and non-HIV SAE microbiome, we discovered that PLWH, had significantly lower bacterial loads, microbial diversity, and species richness compared to HIV- controls. While this observation may seem contrary to what one would expect in a disease associated with immunosuppression and frequent pulmonary infections, it is in fact consistent with numerous microbiome studies comparing disease and non-disease states. Specifically, regardless of the organ examined, disease states are often associated with lower microbial diversity, suggesting that a certain degree of diversity is a hallmark of healthy tissue [24–27]. In the lung, for instance, chronic respiratory conditions such as cystic fibrosis [28, 29] and COPD [30] are associated with lower alpha diversity in sputum and lung samples. Disease severity in the lung also appears to track inversely with diversity [30–32]. In similar fashion, HIV and/or the repeated antibiotic exposures these patients may have experienced due to frequent infections may lower SAE diversity. Supporting this theory are the recent findings by Twigg et al. that patients with uncontrolled HIV have significantly decreased BAL diversity compared with HIV-uninfected controls [6].

The connection between decreased diversity and dysbiosis to COPD pathogenesis is likely multifactorial. In fact, this relationship may be different between HIV+ and HIV- groups, with only the latter showing a correlation between decreased diversity and reduced FEV1/FVC. We were unable to demonstrate significant microbiome differences between patients with and without COPD, regardless of their HIV status. One possible explanation may be that our study was underpowered to detect any difference. Larger studies examining the relationship between airflow obstruction, HIV, and the microbiome are warranted. Targeted brushings in areas of advanced emphysema compared with brushings taken from normal lung within the same PLWH may also help us understand what role the microbiome may play in COPD pathogenesis. Linking the microbiome with metabolomic, transcriptomic, and epigenetic modifications will provide additional clues as to how dysbiosis can set the stage for progressive airflow obstruction. In a previous study by our group looking specifically at the SAE microbiome and its associated transcriptome in HIV [10], we demonstrated that the abundance of Firmicutes was negatively correlated with the expression of cilia-related genes and positively correlated with the expression of immune response genes. *Haemophilus* species were also negatively correlated with the expression of cilia-related genes. These genetic pathways could be critical in the pathogenesis of accelerated COPD in HIV; however, direct comparisons of these relationships to those observed in HIV- subjects are further required.

Interestingly, the SAE phyla distribution in PLWH was markedly different from that in HIV- controls in a pattern that was reminiscent of the differences previously noted between COPD and non-COPD lungs in an HIV- cohort. We found that PLWH had an increase in Proteobacteria and decreases in both Bacteroidetes and Firmicutes compared with HIV- controls. Similarly, Sze et al. found that lung tissue from GOLD Stage 4 COPD patients had increased Proteobacteria and decreased Bacteroidetes and Firmicutes compared to control lungs [30]. For PLWH, changes in the abundance of these three phyla may represent an early stage in the COPD development. Longitudinal studies evaluating the progression of phyla distribution from healthy to diseased lungs may help to clarify this association.

A novel aspect of this study was the use of SAE cells to investigate the unique lung microbiome in HIV. Previous studies have largely focused on BAL fluid, a useful compartment with which to identify generalized inflammation in the lung but not one that necessarily provides specific information on the pathogenesis of COPD. Profound structural changes occur in the SAE in COPD, including squamous metaplasia, ciliary dysfunction, mucous cell hyperplasia, and the breakdown of apical junctional barriers [8]. Even prior to the onset of overt COPD, smoking-related changes in the airway epithelium can be observed. These include senescent signatures determined by telomere length and growth differential factor 15 production [33, 34] and gene expression alterations along immunity and oxidative stress pathways [35]. SAE changes in HIV have not yet been fully characterized, although two studies have recently shed greater light. In one study, the presence of X4 tropic HIV increased both epithelial cell layer permeability and the expression of pro-inflammatory cytokines [36]. In another study, HIV was found to bind to airway epithelial basal cells, resulting in a tissue-destructive phenotype [37]. Whether or not the distinct microbiome of the HIV SAE plays a role in these processes is certainly a question worth pursuing in future experiments.

There are a number of limitations noted in our study. First, contamination by oropharyngeal and environmental elements is always a concern in a lung microbiome study in which specimens are obtained via bronchoscopy. This is particularly true for organs with relatively low microbial biomass such as the lung. Ideally, reagent samples and oral and bronchoscope channel washes prior to the procedure would have helped to identify potential contaminants in bronchial brushings [38]. Nonetheless, all bronchoscopies were performed with no suction used upon insertion of the bronchoscope to avoid oral and large airway contamination. Second, patients enrolled had other pulmonary concerns, including lung masses, nodules, and pneumonia. While these could conceivably pose as confounders, we did not find significant diversity differences between those with and without these conditions. This was likely due to the fact that sample acquisition took place specifically in lobes of the lung away from clinically important lesions. Further studies evaluating the microbiome in asymptomatic PLWH will be necessary moving forward to confirm our findings.

## Conclusions

As the demographics of PLWH shift towards older ages, the spectre of chronic lung diseases looms large and improved understanding of their pathogenesis in the setting of HIV will become imperative. Dysbiosis in the HIV SAE marked by significantly reduced microbial diversity may be one important hallmark or instigator of chronic lung diseases such as COPD and further explorations of the microbiome’s role in this process are warranted.

## Additional file

Additional file 1: **Figure S1.** A rarefaction curve is shown for HIV- subjects (blue) and HIV+ subjects (red). Each line represents a study subject. **Figure S2.** Shannon diversity is shown for the HIV+ group divided by indication for bronchoscopy (*n* = 18 for lung nodule, *n* = 10 for pneumonia). **Figure S3.** Shannon diversity is shown for the HIV+ group comparing those were diagnosed with lung cancer (blue, *n* = 9) and those without lung cancer (red, *n* = 21). **Figure S4.** Shannon diversity is shown for the HIV+ group comparing those who were taking inhaled corticosteroids (blue, *n* = 4) and those who were not (red, *n* = 24). **Figure S5.** Shannon diversity is shown for the HIV+ group comparing those who were taking treatment dose antibiotics (blue, *n* = 8) and those who were not (red, *n* = 20). **Figure S6.** Shannon diversity is shown for the HIV+ group comparing those who were taking treatment prophlactic antibiotics (blue, *n* = 3) and those who were not (red, *n* = 25). (DOCX 651 kb)

## Abbreviations

ART: Antiretroviral therapy
BAL: Bronchoalveolar lavage
COPD: Chronic obstructive pulmonary disease
CT: Computed tomography
FEV1: Forced expiratory volume in 1 s
FVC: Forced vital capacity
GOLD: Global Initiative for Chronic Obstructive Lung Disease
HIV: Human immunodeficiency virus
OTU: Operational taxonomic unit
PCR: Polymerase chain reaction
PLWH: Persons living with HIV
SAE: Small airway epithelium
UBC: University of British Columbia
