# Supplementary material for: Decreased microbiome diversity in the HIV small airway epithelium
Source: Respir Res. 2018 Jul 27;19:140. doi: 10.1186/s12931-018-0835-7 (PMC6062954; doi:10.1186/s12931-018-0835-7)
Supplement: Supplementary file 1 — Figure S1. A rarefaction curve is shown for HIV- subjects (blue) and HIV+ subjects (red). Each line represents a study subject. Figure S2. Shannon diversity is shown for the HIV+ group divided by indication for bronchoscopy (n = 18 for lung nodule, n = 10 for pneumonia). Figure S3. Shannon diversity is shown for the HIV+ group comparing those were diagnosed with lung cancer (blue, n = 9) and those without lung cancer (red, n = 21). Figure S4. Shannon diversity is shown for the HIV+ group comparing those who were taking inhaled corticosteroids (blue, n = 4) and those who were not (red, n = 24). Figure S5. Shannon diversity is shown for the HIV+ group comparing those who were taking treatment dose antibiotics (blue, n = 8) and those who were not (red, n = 20). Figure S6. Shannon diversity is shown for the HIV+ group comparing those who were taking treatment prophlactic antibiotics (blue, n = 3) and those who were not (red, n = 25). (DOCX 651 kb) [file 12931_2018_835_MOESM1_ESM.docx]

**Decreased Microbiome Diversity in the HIV Small Airway Epithelium**

**Additional file 1**

**Authors**

Stella Xu

Amy Tsai

Marc A Sze

Emily A Vucic

Tawimas Shaipanich

Marianne Harris

Silvia Guillemi

Julia Yang

Sunita Sinha

Corey Nislow

Julio Montaner

Wan Lam

Stephen Lam

Don D Sin

S F Paul Man

Janice M Leung

**Supplemental Methods**

Droplet Digital PCR

To quantify total bacteria load in each sample, primers specifying the 293bp amplicon of the 16S rRNA gene (Table S3 in Appendix C) were designed using the protocol outlined by Sze et al.^1^ The experiment was performed using a SYBR green based assay with the Bio-Rad QX200^TM^ system. For each 20 ul PCR reaction, 10 ul of 2X ddPCR Supermix for Probes (Bio-Rad, Catalog #186-3010), 0.1 uM 63F primer, 0.1 uM 355R primer, 2 ul of DNA template and 7.6 ul of distilled water were used. The PCR cycling conditions were 1 cycle at 95°C for 5 minutes, 40 cycles at 95°C for 30 seconds and 60°C for 1 minute, 1 cycle at 4°C for 5 minutes and 1 cycle at 90°C for 5 minutes. All cycles were run at a ramp speed of 2°C per second. The PCR reaction was performed with a Bio-Rad T100 thermal cycler and quantified using Bio-Rad Quantisoft software. A threshold cutoff of 20,000 that effectively separates a positive signal from a negative signal was chosen based on preliminary experiments.

MiSeq Sequencing Pipeline

A pooled library consisting of all the samples with individually labeled indices were generated using the protocol adopted from a dual-index sequencing strategy published by Kozich et al.^2^ The first step in the pipeline was to perform a touchdown PCR. For each 20ul PCR reaction, 2 ul of 10X AccuPrime PCR Buffer II, 0.15 ul of AccuPrime Taq DNA Polymerase High Fidelity, 2 ul of forward and reverse primer mixture at 0.5 uM each, 2 ul of DNA template, and 13.85 ul of distilled water were used. Each primer sequence was individually labeled with unique index sequences for identification. The cycling conditions of touchdown PCR was adapted from the protocol of Korbie & Mattick: 1 cycle at 95°C for 2 minutes, 20 cycles at 95°C for 2 minutes 60°C /54°C for 15 seconds and 72°C for 90 seconds (temperature descend at 0.3°C per cycle), 20 cycles at 95°C for 20 seconds 55°C for 15 seconds and 72°C for 90 seconds, 1 cycle at 72°C for 5 minutes.^3^ The touchdown PCR amplified 16S rRNA gene fragments spanning the V4 region. After the PCR, products were purified with Agencourt AMPure XP system (Beckman Coulter, Catalog #A63880) following the manufacturer’s protocol. Samples were randomly selected for quality check using gel electrophoresis. All tested samples had one single strong band which indicated they were qualified to be pooled. The library pool was generated using the SequalPrep^TM^ normalization plate (Thermo Fisher Scientific, Catalog #A1051001). 25 ul of purified PCR product was transferred to the plate and sequentially eluted in 20 ul of elution buffer. DNA quality and quantity check was carried out using Agilent high sensitivity DNA kit (Agilent, Catalog #5067-4626). Lastly, sequencing was performed on the Illumina MiSeq^TM^ platform (Illumina, Redwood City, CA, USA) with 2 x 250 paired end-read chemistry at the UBC Sequencing and Bioinformatics Consortium.

References

1. Sze MA, Abbasi M, Hogg JC, Sin DD. A comparison between droplet digital and quantitative PCR in the analysis of bacterial 16S load in lung tissue samples from control and COPD GOLD 2. *PLoS One*. 2014. doi:10.1371/journal.pone.0110351.
2. Kozich JJ, Westcott SL, Baxter NT, Highlander SK, Schloss PD. Development of a dual-index sequencing strategy and curation pipeline for analyzing amplicon sequence data on the miseq illumina sequencing platform. *Appl Environ Microbiol*. 2013;79(17):5112-5120. doi:10.1128/AEM.01043-13.
3. Korbie DJ, Mattick JS. Touchdown PCR for increased specificity and sensitivity in PCR amplification. *Nat Protoc*. 2008;3(9):13-15. doi:10.1038/nprot.2008.133.

**Figure S1**


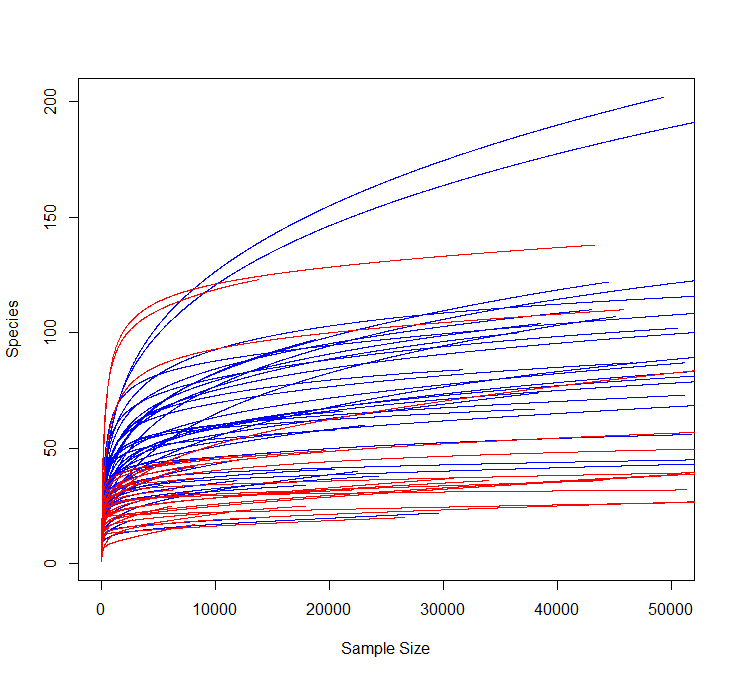


A rarefaction curve is shown for HIV- subjects (blue) and HIV+ subjects (red). Each line represents a study subject. The number of sequences identified by random sampling is represented on the x-axis while the y-axis represents the number of species found by random sampling. Greater richness was therefore found in the HIV- subjects compared to the HIV+ subjects.

**Figure S2**

**A.**


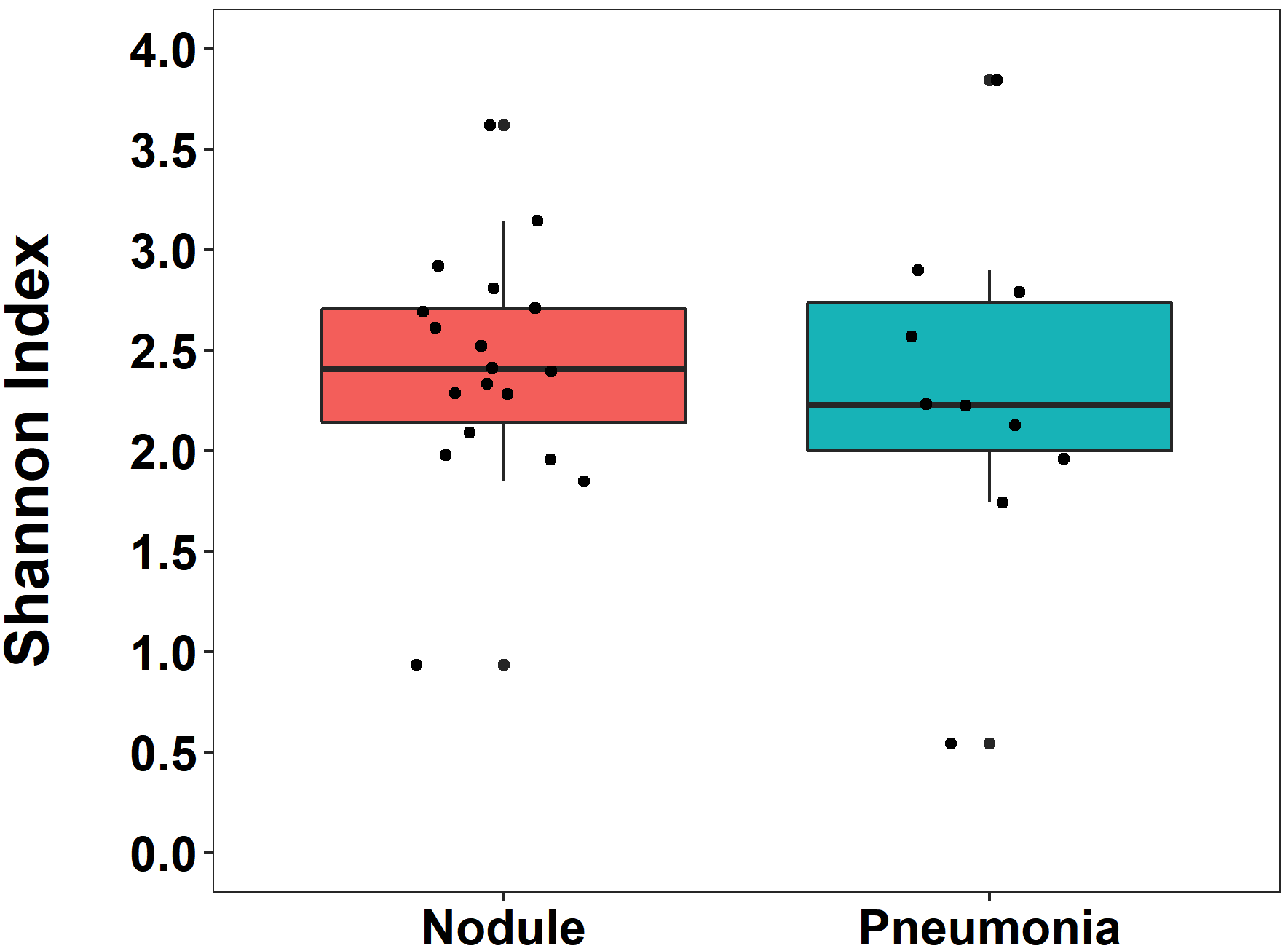


Shannon diversity is shown for the HIV+ group divided by indication for bronchoscopy (n=18 for lung nodule, n=10 for pneumonia). There was no significant difference in Shannon diversity between these two groups (p=0.55).

**B.**


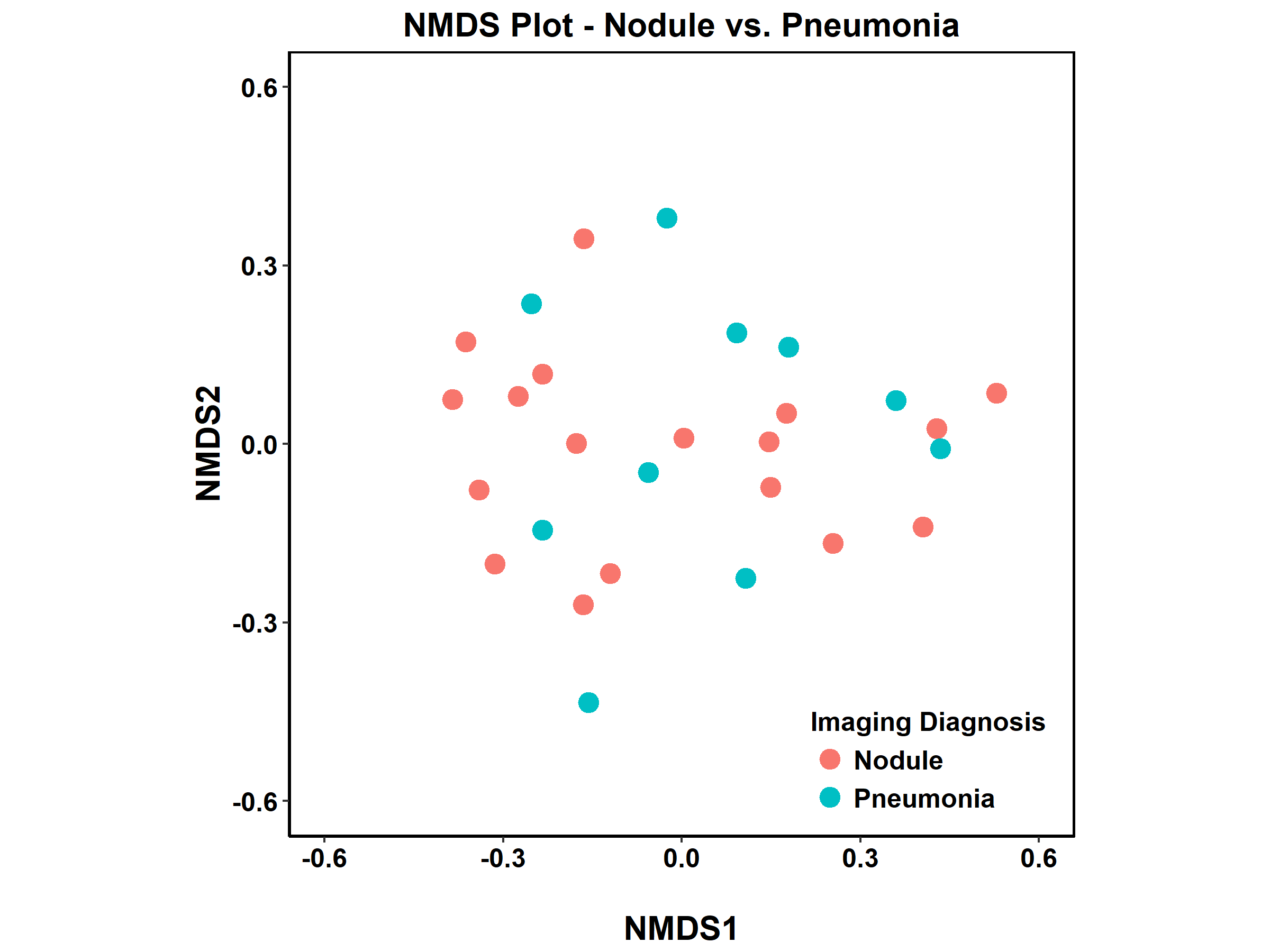


NMDS plot is shown for the HIV+ group divided by indication for bronchoscopy. There was no significant difference in community composition between these two groups (PERMANOVA=0.061).

**Figure S3**

**A.**

**
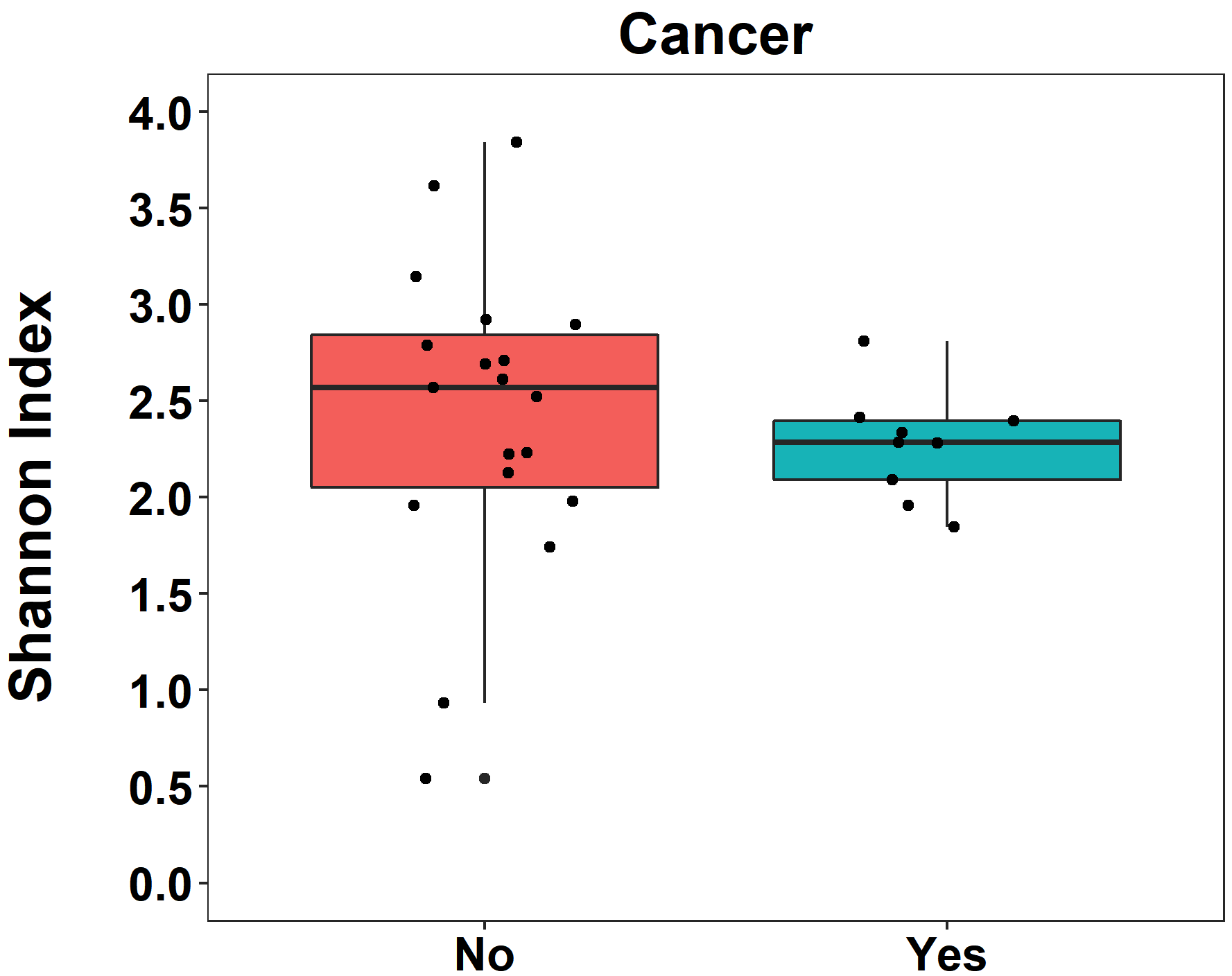
**

Shannon diversity is shown for the HIV+ group comparing those were diagnosed with lung cancer (blue, n=9) and those without lung cancer (red, n=21). There was no significant difference in Shannon diversity (p=0.33).

**B.**

**
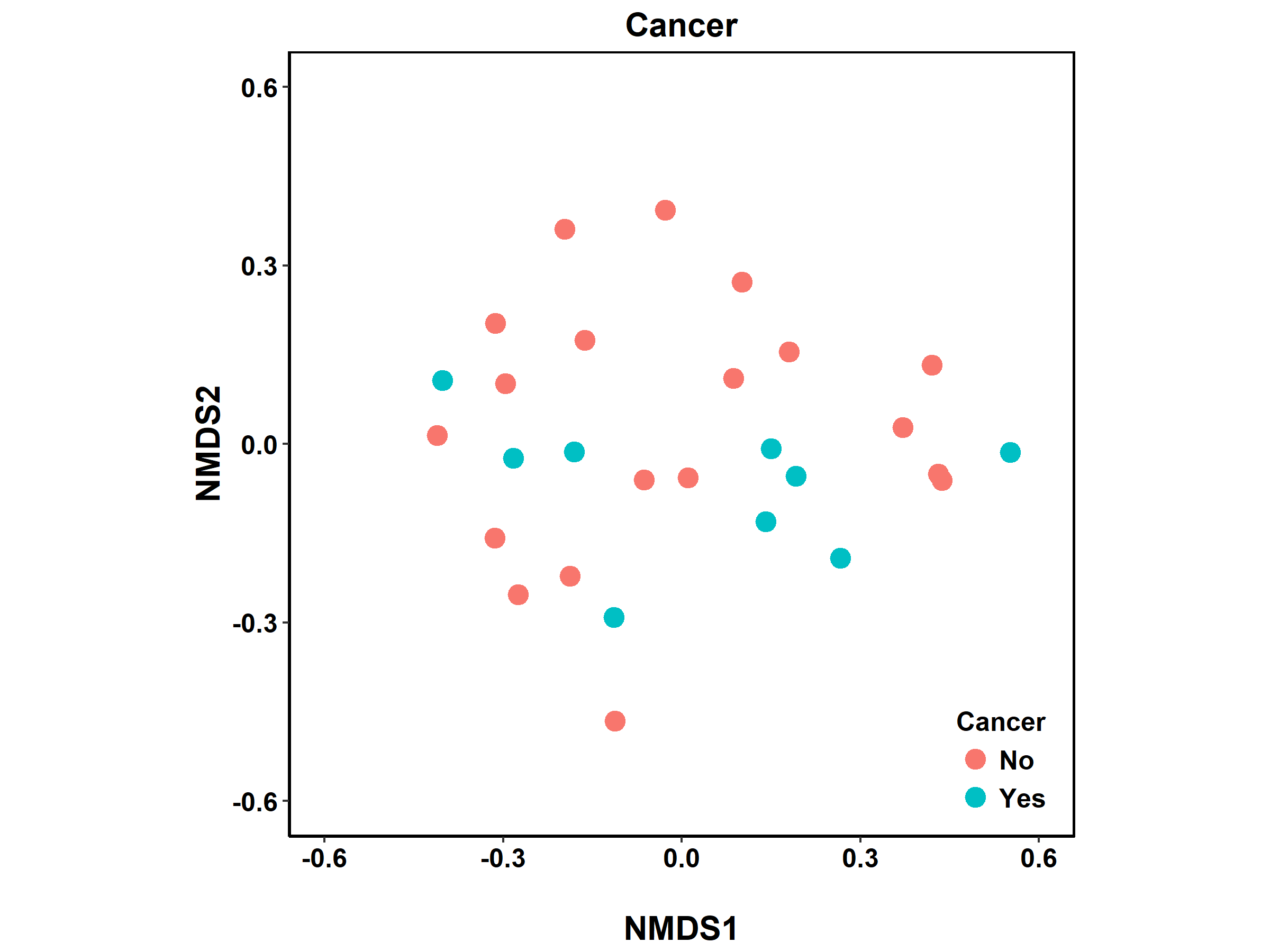
**

NMDS plot is shown for the HIV+ group divided by presence of lung cancer. There was no significant difference in community composition (PERMANOVA=0.66).

**Figure S4**

**A.**


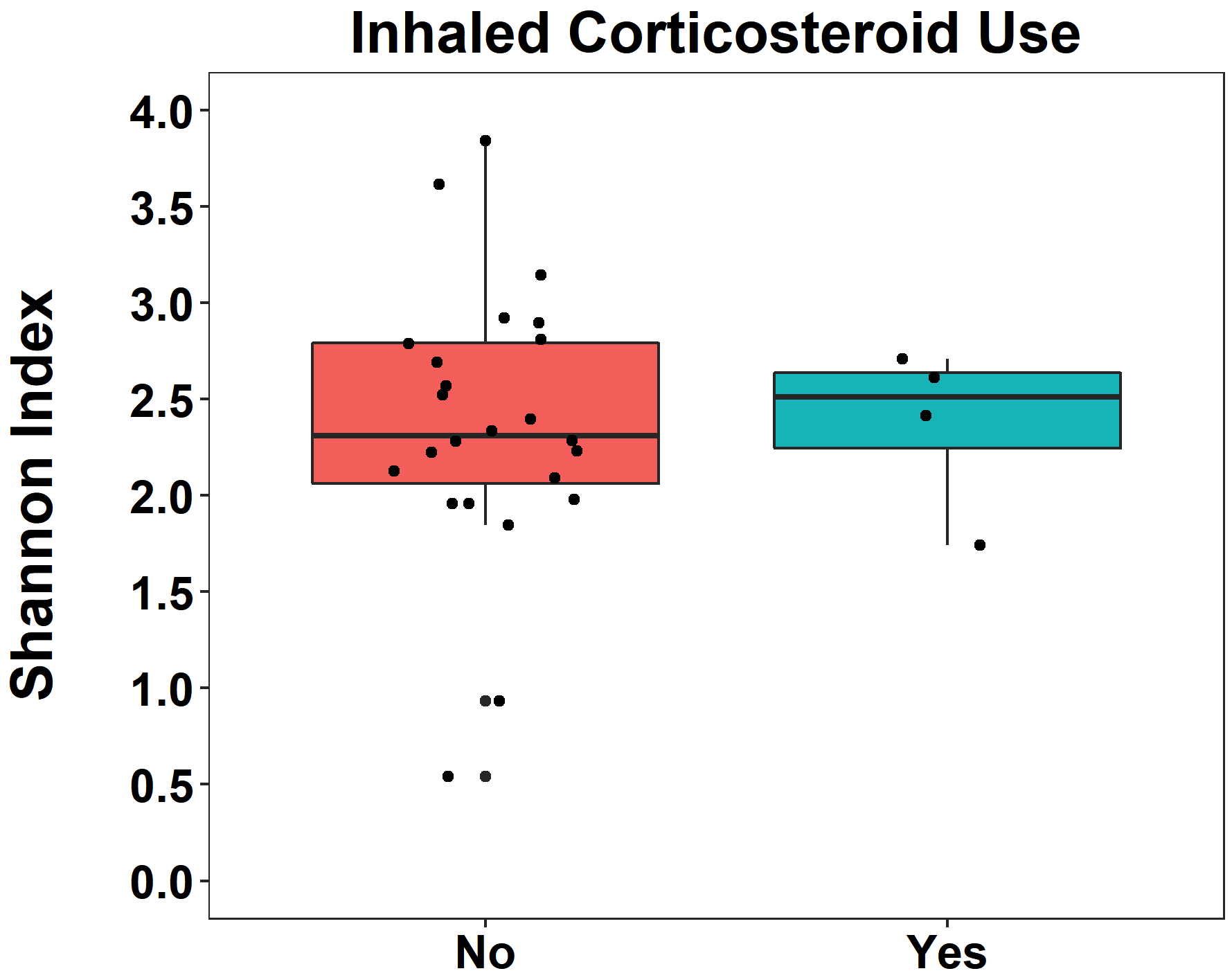


Shannon diversity is shown for the HIV+ group comparing those who were taking inhaled corticosteroids (blue, n=4) and those who were not (red, n=24). There was no significant difference in Shannon diversity (p=0.97).

**B.**

**
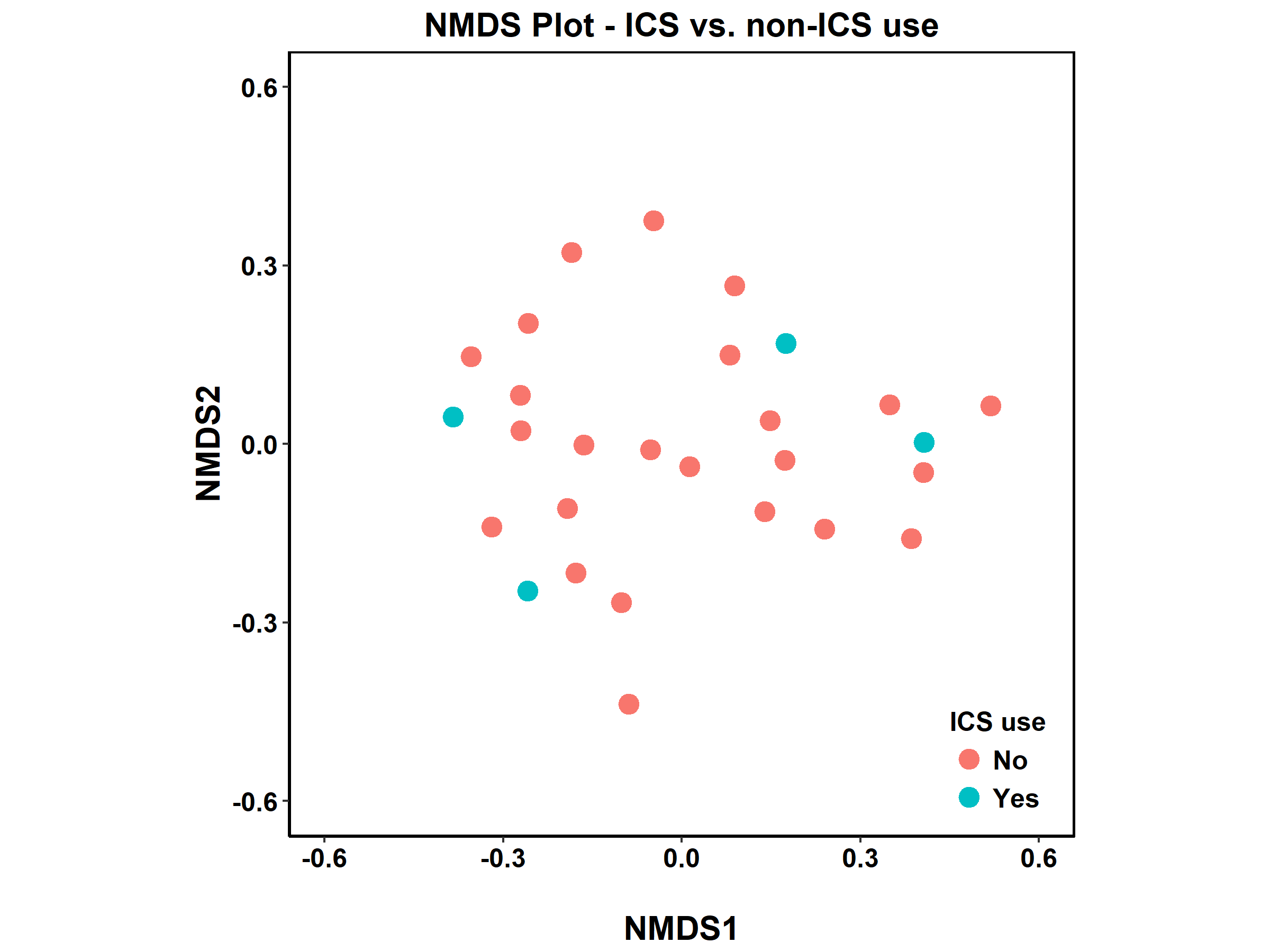
**

NMDS plot is shown for the HIV+ group divided by use of inhaled corticosteroids. There was no significant difference in community composition (PERMANOVA=0.99).

**Figure S5**

**A.**

**
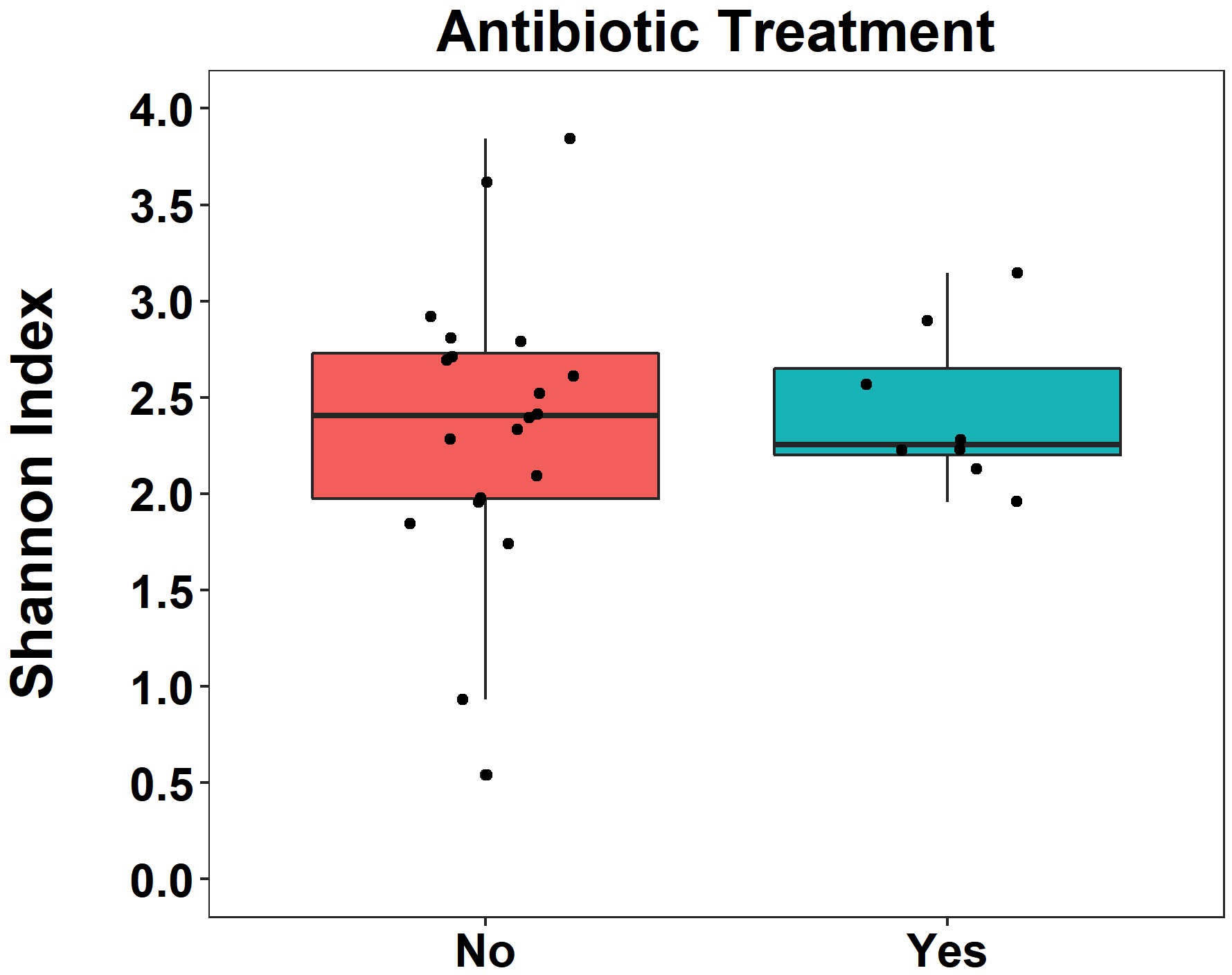
**

Shannon diversity is shown for the HIV+ group comparing those who were taking treatment dose antibiotics (blue, n=8) and those who were not (red, n=20). There was no significant difference in Shannon diversity (p=1.0).

**B.**


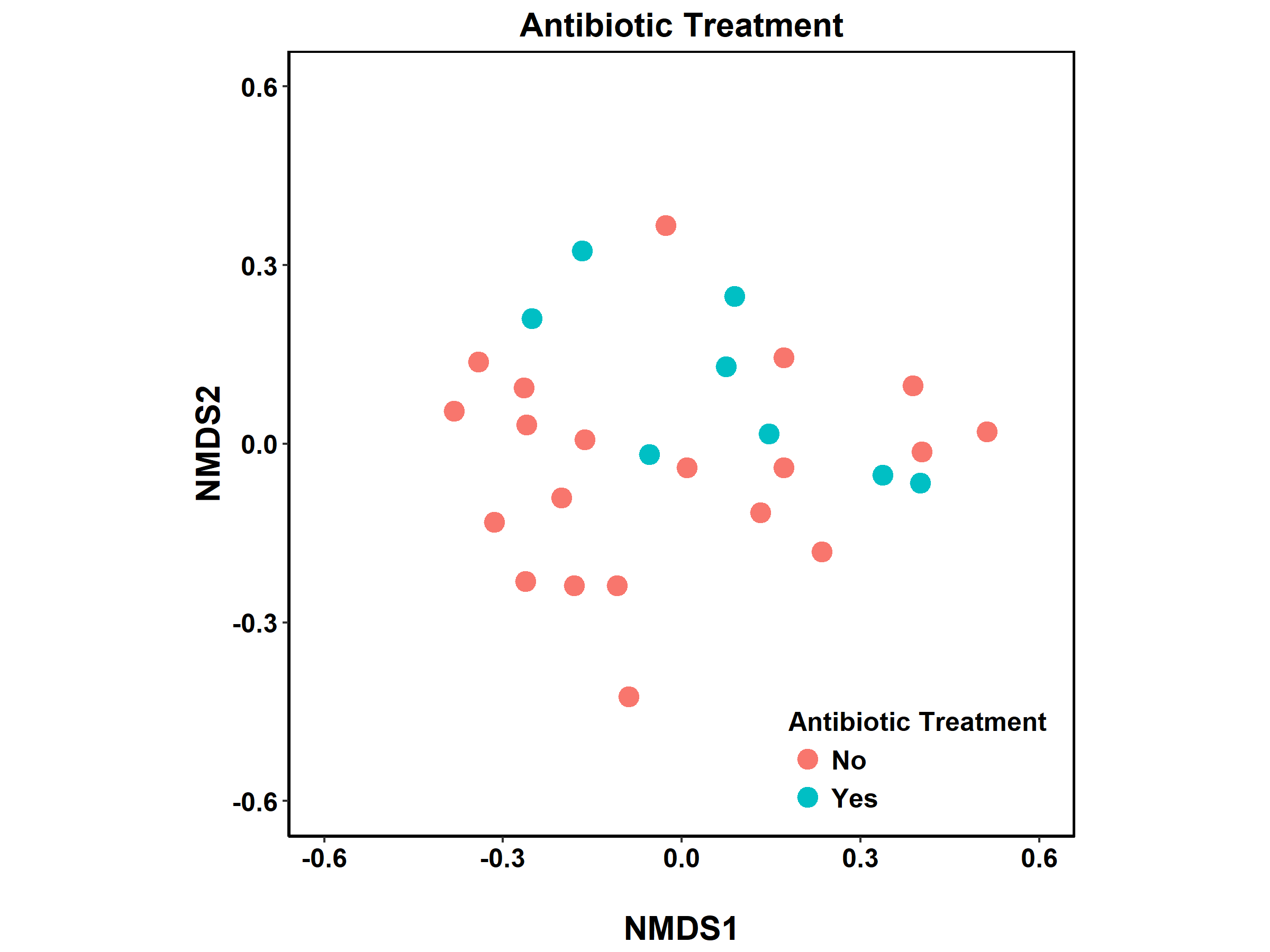


NMDS plot is shown for the HIV+ group divided by use of treatment antibiotics. There was no significant difference in community composition (PERMANOVA=0.73).

**Figure S6**

**A.**

**
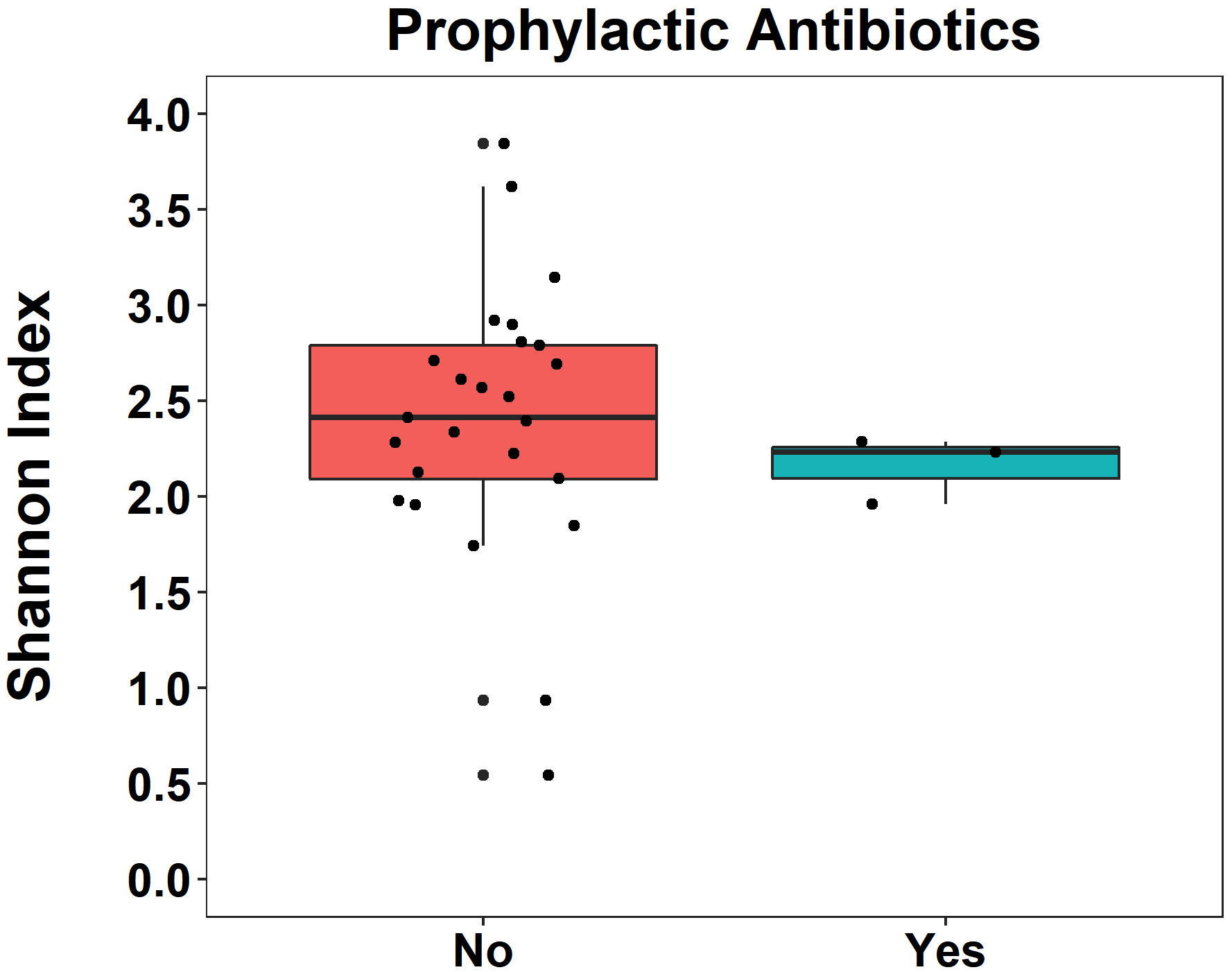
**

Shannon diversity is shown for the HIV+ group comparing those who were taking treatment prophlactic antibiotics (blue, n=3) and those who were not (red, n=25). There was no significant difference in Shannon diversity (p=0.35).

**B.**

**
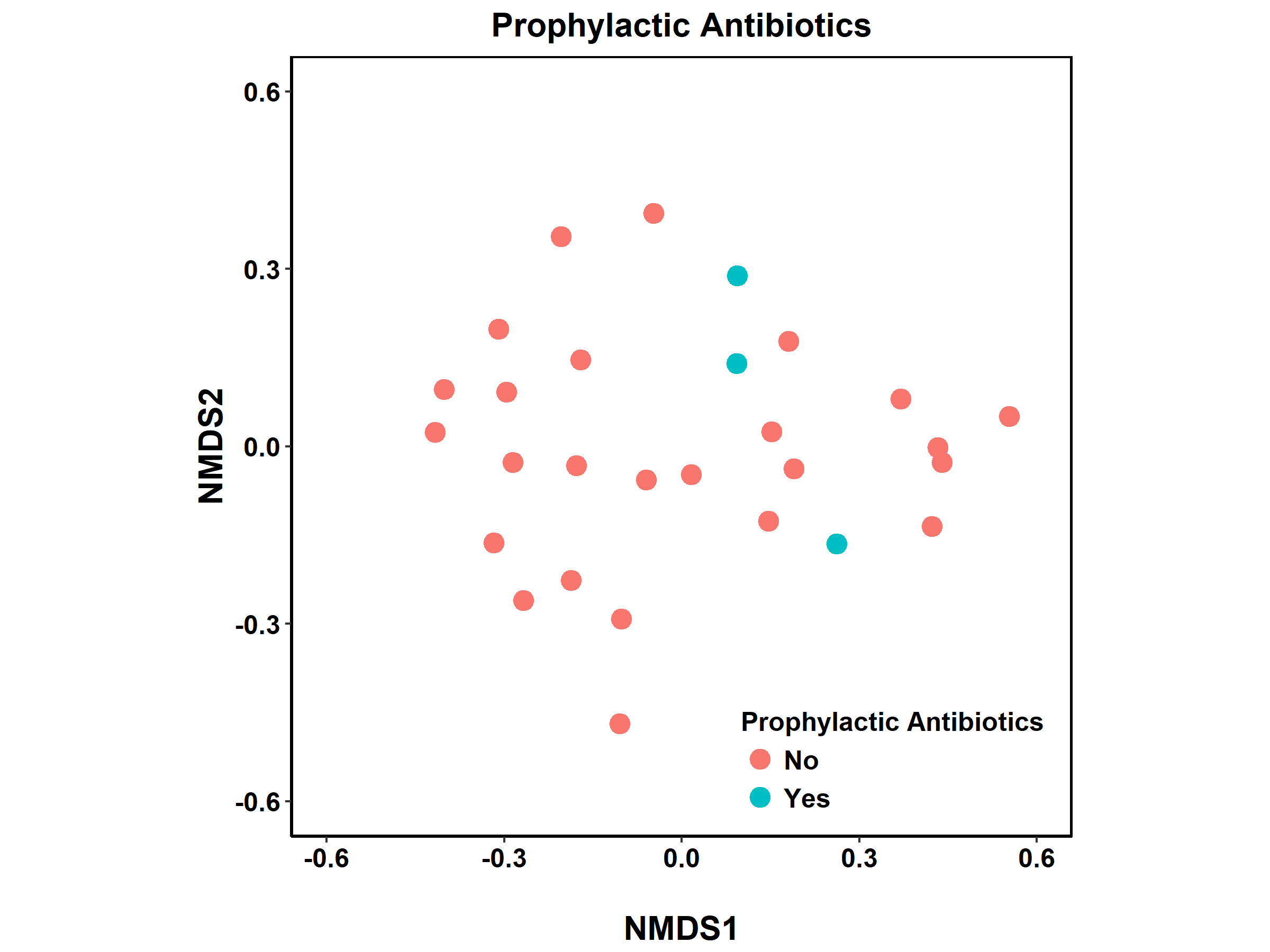
**

NMDS plot is shown for the HIV+ group divided by use of prophylactic antibiotics. There was no significant difference in community composition (PERMANOVA=0.73).
